# Organoid-based personalized medicine: from tumor outcome prediction to autologous transplantation

**DOI:** 10.1093/stmcls/sxae023

**Published:** 2024-03-25

**Authors:** Abel Soto-Gamez, Jeremy P Gunawan, Lara Barazzuol, Sarah Pringle, Rob P Coppes

**Affiliations:** Department of Biomedical Sciences, University of Groningen (RUG) and University Medical Center Groningen (UMCG), Groningen, The Netherlands; Department of Radiation Oncology, University of Groningen (RUG) and University Medical Center Groningen (UMCG), Groningen, The Netherlands; Department of Biomedical Sciences, University of Groningen (RUG) and University Medical Center Groningen (UMCG), Groningen, The Netherlands; Department of Radiation Oncology, University of Groningen (RUG) and University Medical Center Groningen (UMCG), Groningen, The Netherlands; Department of Biomedical Sciences, University of Groningen (RUG) and University Medical Center Groningen (UMCG), Groningen, The Netherlands; Department of Radiation Oncology, University of Groningen (RUG) and University Medical Center Groningen (UMCG), Groningen, The Netherlands; Department of Rheumatology and Clinical Immunology, University of Groningen (RUG) and University Medical Center Groningen (UMCG), Groningen, The Netherlands; Department of Biomedical Sciences, University of Groningen (RUG) and University Medical Center Groningen (UMCG), Groningen, The Netherlands; Department of Radiation Oncology, University of Groningen (RUG) and University Medical Center Groningen (UMCG), Groningen, The Netherlands

**Keywords:** organoids, patient-derived organoids, personalized medicine, cell-based therapy

## Abstract

Inter-individual variation largely influences disease susceptibility, as well as response to therapy. In a clinical context, the optimal treatment of a disease should consider inter-individual variation and formulate tailored decisions at an individual level. In recent years, emerging organoid technologies promise to capture part of an individual’s phenotypic variability and prove helpful in providing clinically relevant molecular insights. Organoids are stem cell-derived 3-dimensional models that contain multiple cell types that can self-organize and give rise to complex structures mimicking the organization and functionality of the tissue of origin. Organoids therefore represent a more faithful recapitulation of the dynamics of the tissues of interest, compared to conventional monolayer cultures, thus supporting their use in evaluating disease prognosis, or as a tool to predict treatment outcomes. Additionally, the individualized nature of patient-derived organoids enables the use of autologous organoids as a source of transplantable material not limited by histocompatibility. An increasing amount of preclinical evidence has paved the way for clinical trials exploring the applications of organoid-based technologies, some of which are in phase I/II. This review focuses on the recent progress concerning the use of patient-derived organoids in personalized medicine, including (1) diagnostics and disease prognosis, (2) treatment outcome prediction to guide therapeutic advice, and (3) organoid transplantation or cell-based therapies. We discuss examples of these potential applications and the challenges associated with their future implementation.

## Graphical Abstract

**Graphical Abstract:**
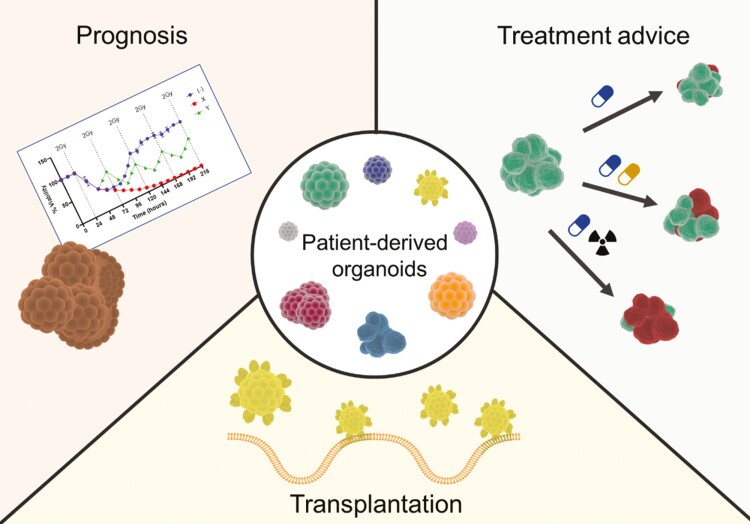

**Significance Statement**

This review summarizes the progress made on using patient-derived organoids in personalized medicine context. We discuss examples of organoid applications such as diagnostics, treatment outcome prediction, and organoid transplantation, as well as exploring the challenges associated with their implementation.

## Introduction

Technological advances have enabled the high-throughput characterization and quantification of biological molecules (DNA, RNA, proteins, lipids, and other metabolites) and their relation to phenotypes such as structure and function. This type of research, referred to as “omics” (e.g., genomics, transcriptomics, proteomics, lipidomics, and metabolomics), has demonstrated a large degree of inter-individual variation in both health and disease. These differences often underlie disease etiology and have the potential to be addressed in the context of personalized medicine.

Personalized medicine postulates that the optimal treatment of a disease should consider inter-individual variation and formulate tailored decisions or solutions at an individual level, based on the subject’s unique profile (reviewed in^1^). However, a key challenge of personalized medicine is developing robust ways to generate these profiles. In this context, it is now possible to create miniature partial organs or “organoids” from an individual’s own cells, which can capture many of an individual’s molecular phenotypes.

**Figure 1.**
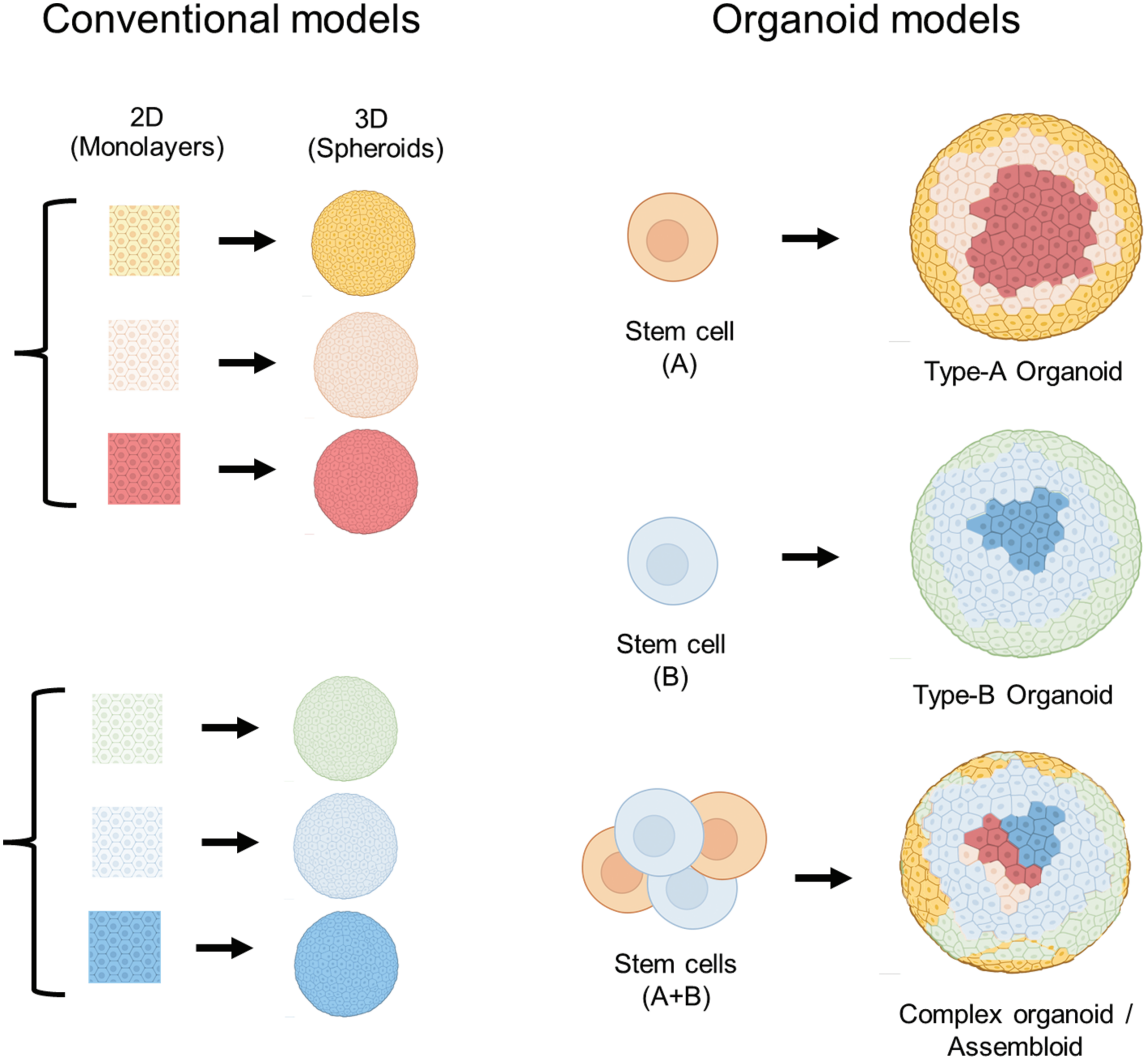
Conventional vs organoid models. Conventional models are typically composed of a single-cell type cultured as monolayers. More recently, the advent of specialized low adhesive coatings or synthetic matrixes has enabled the transition to 3D, often spherical structures, termed spheroids. Spheroids account for cell-cell interactions but lack cell type diversity and structure. In contrast, organoid models are stem cell-derived structures that can give rise to multiple cell types and structures of the same tissue. The combination of stem cells from different lineages under specific conditions can give rise to even more complex organoids often termed “assembloids” capable of modeling multi-lineage interactions, and potentially yield improved higher complexity models.

Organoids are stem cell-derived 3-dimensional structures that resemble their donor tissue of origin.^2^ Organoids can serve as in vitro models with higher complexity than conventional 2-dimensional cultures, and unlike conventional cells grown as monolayers, organoids contain multiple cell types able to self-organize and give rise to complex structures that mimic the function of the tissue of origin (Fig. 1). Because of these properties, organoids hold promise of providing greater understanding of molecular pathologies, offer insights into disease prognosis, and even predict treatment outcomes. The most studied application of organoids is the modelling and optimization of possible cancer treatments, attributable to the influence of cancer heterogeneity on the success of treatment outcome. In this setting, tumor-derived organoids, also termed tumoroids, represent important sources of variation that could be useful for modeling heterogeneity in treatment response.^3^

In addition, the individualized nature of patient-derived normal tissue organoids also opens the possibility of using them as autologous sources of transplantation material. This personalized medicine approach could yield biological material expanded from the same patient, and is thereby not limited by histocompatibility. An increasing amount of preclinical evidence has now paved the way for ongoing organoid-based clinical trials currently under investigation. This review will focus on the clinical progress made so far concerning the use of organoids in personalized medicine, as well as the many challenges associated with their future implementation. Following this, we will discuss the properties that make organoid technologies better suited than conventional culture methods.

### Cell type diversity

Traditional cell culture models have been pivotal to the current understanding of our complex biology in both health and disease. However, 2D cell cultures lack many crucial features necessary for their application in personalized medicine. For instance, cellular monolayers cultured from primary tissue often lack cellular heterogeneity and are composed mostly of a single-cell type (Fig. 1). When cultured in 3D format, cell lines may form structures, often spherical (termed “spheroids”) which account for cell-cell interactions but lack cell type diversity and structure. These models therefore cannot recapitulate cell-cell interactions across cell types, many of which are important in certain disease contexts such as, eg, the (mis)-communication between immune cells and normal tissues, or the stem cell crosstalk and differentiation process in response to injury. Organoid models hold promise of revealing key interactions between different cell types, and are used in the identification of novel molecular mechanisms and drug targets^4-7^ (reviewed in Refs. ^1^ and ^2^).

Organoid models are derived from stem cells that have the capacity to give rise to multiple cell types of the same tissue.^2^ However, despite increased cellular diversity, most existing organoid models lack multi-lineage interactions between cell types derived from different lineages (eg, mesoderm-derived immune cells and ectoderm-derived neurons). These limitations have prompted the development of so-called assembloids (Fig. 1), where cells of different lineages are aggregated together and are able to self-organize giving rise to more complex organoids containing multi-lineage interactions depending on the initial constituents added.^8,9^ Alternatively, coculture models where organoids are separated by semi-permeable membranes from cell types of other lineages (eg, epithelial-derived organoids and mesenchymal stem cells) enable the study of ligand-receptor interactions, without accounting for membrane-based cell-cell interactions.^10,11^

### Cell-cell interactions

In addition to cell-type diversity, 3D organoids may also recapitulate cell-cell interactions between cell types, such as those mediated by tight junctions, anchoring junctions, or gap junctions. Cell-cell interactions promote more faithful cell polarities and morphologies which are often lacking in cell line-derived 2D monolayers. Organoids are therefore capable of modeling complex cellular behavior such as self-organization and 3D contact inhibition. In 2D monolayers contact inhibition stops cells from dividing after reaching high densities.^12^ In organoid models contact inhibition is much more complex, not inhibited by mere cell-cell contact, but influenced by the interacting cell types and their relative position (eg, basal and luminal cell interactions^13^). These features of normal cells are frequently lost in cancer but are essential for proper development and tissue repair. The use of organoid models could therefore be useful for understanding the molecular mechanisms behind these processes.

### Cell-matrix interactions

The extracellular matrix (ECM) is a network of fibrous proteins and glycoproteins that surround cells and provide them with tissue-specific biochemical and mechanical cues (reviewed in Ref. ^14^). The composition and architecture of the ECM are typically organ specific and may play a role in defining cell identity, morphology, and polarity. For instance, the presence or the absence of ECM may be used to control epithelial polarity, coercing organoids to display the apical surface outward in order to model specific biology (eg, lipid uptake, bacterial infection^15^). Much of the ECM used for organoid formation is derived from basement membrane extracts of the Engelbreth-Holm-Swarm tumor (BME/Matrigel). The extract is mainly composed of laminin, type IV collagen, heparan sulfate proteoglycan, entactin, and nidogen.^16^ These components polymerize to form a biologically active hydrogel matrix used as a substrate for 3D cultures.^17^

One of the limitations for the use of Matrigel in the clinic is that it is poorly defined and can differ in batch-to-batch consistency. Alternative options include the use of defined substrates (eg, polyethylene-glycol, PEG; nanocellulose, alginate, and hyaluronic acid), which offer the advantage of being chemically defined and are often more affordable (reviewed in Ref. ^18^). However, synthetic hydrogels do not always possess the same growth promoting properties as Matrigel, such as not supporting extensive self-renewal and promoting stem cell differentiation (^19,20^). Recent efforts have yielded the first fully synthetic matrices supporting serial passaging of epithelial organoids,^21,22^ but their adoption by more research groups and their usage in broader organoid types remains to be determined.

### Stem cell origin

Organoid technology relies on the stem cell’s capacity of self-renewal and multipotency. There are 2 approaches to generate organoids, from adult stem cells or pluripotent stem cells. Adult stem cells (ASCs) are undifferentiated somatic stem cells that reside within adult organ tissues. Meanwhile, pluripotent stem cells are found in the blastocyst stage of embryonic development. It is now possible to reprogram differentiated human adult cells back into induced pluripotent stem cells (iPSCs).^23^ Under the right conditions of growth factors, PSCs could generate organoids which remain unfeasible using ASCs such as the brain, retina, and blood vessels.^24^

Meanwhile, cancer tissue has been established to host populations of heterogenous and proliferative cells that can generate tumor-like organoids, or “tumoroids” which mimic primary tumor tissues in histopathology, genetic profiles, and mutations.^25^ Most importantly, they are known to faithfully replicate the therapeutic response of the sampled tumor to chemo- and radiotherapy.^3,26,27^ Together, these tumoroids are a valuable means to study cancer prognosis and deliver treatment advice. However, tumoroids can be difficult to produce as primary tumor tissues may also contain healthy ASCs, potentially generating non-malignant organoids outcompeting the growth of tumoroids, as observed in non-small cell lung cancer and prostate cancer organoids.^28^ Therefore, tumoroids must be characterized thoroughly before use, as purity can become a hurdle during assays needed in precision medicine.

## Personalized medicine applications

The establishment of organoid biobanks represents a crucial step in the use of organoid technology for personalized medicine. Living organoid biobanks, a repository of characterized and cultivated organoids, are now available for a variety of tissues.^26,29,30^

A plethora of organoid types derived from humans as well as animal models has been developed and extensively characterized. Many of these have demonstrated innovative applications in preclinical contexts, with remarkable promissory outcomes. However, very few of these have successfully transitioned into clinical evaluation.

**Figure 2.**
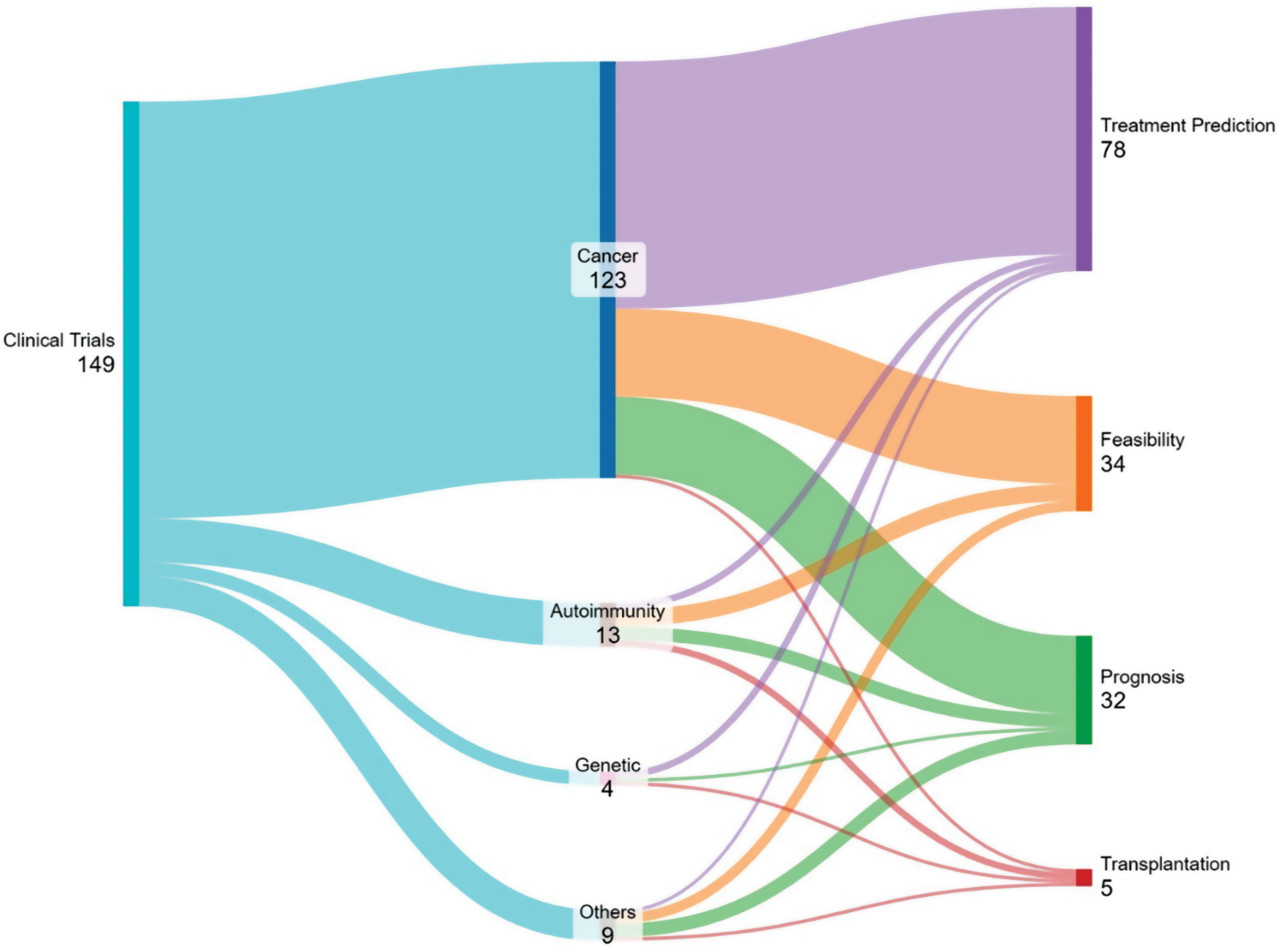
Organoid-based clinical trials. Sankey chart showing the distribution of registered clinical trials making use of organoid technologies as of November 2023 (right). The number of trials is segregated in terms of disease context (middle) and intended clinical application (left).

In our review, we focus on how organoid technology is presently being evaluated in the clinic for personalized medicine applications, particularly in (1) diagnosis and disease prognosis, (2) treatment advice, and (3) organoid stem cell-based therapeutics, in the context of ongoing clinical trials. A search for “observational” and “interventional” clinical trials in the US registry (clinicaltrials.gov) using the search term “organoid(s)” revealed that almost 85% of registered trials focus on different types of cancer or cancer-related outcomes (Fig. 2). Other clinical trials start to emerge focusing on autoimmune diseases or genetic disorders (10%), while every other disease-type grouped together constitutes the rest of all indications (5%). Furthermore, half of all registered trials focus on treatment outcome prediction (52%), intended to guide oncologists in optimizing a therapeutic strategy for the treatment of diverse cancers.

Importantly, a large variety of tissue-type organoids have already been developed and are now being used in clinical contexts (Table 1). Among these, gastrointestinal organoids are the most common tissue type under examination, perhaps due to being the first type of organoid being developed and described in 2009 by the Clevers’ group.^2^ Intestinal organoids have since been extensively studied and further developed by the Clever’s group and many other research groups all over the world. The high level of characterization of intestinal organoids has warranted their exploration in other areas beyond cancer, such as autoimmune diseases (IBD, Chron’s disease), dietary intolerances, and even as autologous sources for transplantation material.

**Table 1.** Types of organoids in clinical trials.

Organoid	Disease	Application	Phase	Trial number	
Brain	Cancer	Feasibility	NA (5)	NCT05772767 NCT05772741 NCT03971812	NCT04478877 NCT04927611
		Prognostic	NA (1)	NCT04868396	
		Guidance	NA (1), Phase 1 (2)	NCT04865315 NCT05432518	NCT05473923
	Genetic (Psychiatric disorders)	Prognostic	NA (1)	NCT05480826	
Breast	Cancer	Feasibility	NA (2), Phase I (1)	NCT05404321 NCT05317221	NCT04727632
		Prognostic	NA (1)	NCT04531696	
		Guidance	NA (11), Phase I (2), Phase II (1), Phase III (1)	NCT04450706 NCT05532397 NCT04281641 NCT05007379 NCT04504747 NCT04526587 NCT01287468 NCT05767931	NCT04655573 NCT02732860 NCT04703244 NCT05177432 NCT05134779 NCT05381038 NCT05429684
Gastrointestinal	Autoimmune (IBD, Chron’s Disease)	Feasibility	NA (3)	NCT05294107 NCT04853212	NCT02710370
		Prognostic	NA (4)	NCT05259826 NCT05056610	NCT02874365 NCT03256266
		Guidance	NA (2)	NCT05425901	NCT03529318
		Transplant	NA (1)	UMIN000030117	
	Cancer	Feasibility	NA (5)	NCT04278326 NCT04371198 NCT00646022	NCT04220242 NCT04896684
		Prognostic	NA (5), Phase II (1)	NCT04842006 NCT03429816 NCT05916443	NCT03874559 NCT05038358 NCT05078866
		Guidance	NA (19), Phase I (1), Phase II (1)	NCT05832398 NCT05352165 NCT05203549 NCT03577808 NCT03283527 NCT04755907 NCT04219137 NCT05442138 NCT05183425 NCT04906733 NCT05883683	NCT04996355 NCT05652348 NCT03081988 NCT05401318 NCT04611035 NCT05304741 NCT05384184 NCT05351398 NCT05630794 NCT05725200
	Other (Food allergies)	Feasibility	NA (2)	NCT05323357	NCT04497727
		Prognostic	NA (1)	NCT02888587	
		Guidance	NA (1)	NCT04549727	
Kidney	Cancer	Feasibility	NA (2)	NCT04497727	NCT05323357
	Other (Chronic kidney disease)	Prognostic	NA (1)	NCT04874909	
Liver	Cancer	Feasibility	NA (3)	NCT03307538 NCT05720676	NCT02436564
		Prognostic	NA (1), Phase II (1)	NCT02718235	NCT04072445
		Guidance	NA (5)	NCT05644743 NCT04561453 NCT05913141	NCT04622423 NCT05634694
Lung	Cancer	Feasibility	NA (1)	NCT05251805	
		Prognostic	NA (3), Phase II (1)	NCT03655015 NCT05092009	NCT04826913 NCT05411107
		Guidance	NA (5), Phase II (1)	NCT05136014 NCT04859166 NCT03453307	NCT05332925 NCT03979170 NCT05669586
	Autoimmune (COPD)	Feasibility	NA (2)	NCT05227547	NCT02705144
	Genetic (Cystic fibrosis)	Guidance	NA (2)	NCT05100823	NCT03390985
Pancreatic	Autoimmune (Type I diabetes)	Transplant	Phase II (1)	NCT03163511	
	Cancer	Feasibility	NA (1), Phase II (1)	NCT03140592	NCT04469556
		Prognostic	NA (2)	NCT05727020	NCT02869802
		Guidance	NA (7), Phase III (2)	NCT05351983 NCT05842187 NCT04777604 NCT03544255 NCT05196334	NCT04736043 NCT05927298 NCT04931394 NCT04931381
Reproductive	Cancer	Feasibility	NA (2)	NCT05537844	NCT04770974
		Guidance	NA (6)	NCT05813509 NCT05175326 NCT05577689	NCT04555473 NCT05290961 NCT04768270
	Others (Endometriosis)	Feasibility	NA (1)	NCT05521932	
		Prognostic	NA (2)	NCT05412771	NCT04939064
Salivary	Others (Radiation complications)	Transplant	NA (1)	NCT04593589	
Retinal	Genetic (Retinitis Pigmentosa)	Transplant	NA (1)	jRCTa050200027	
Others (Solid tumors, soft tissue)	Cancer	Feasibility	NA (3)	NCT04261192 NCT05786144	NCT04714957
		Prognostic	NA (6), Phase II (1)	NCT02910895 NCT05734963 NCT04723316 NCT05375266	NCT05918510 NCT05696002 NCT03146962
		Transplant	Phase I (1)	NCT03778814	
		Guidance	NA (7), Phase II (1)	NCT05267912 NCT05400239 NCT03890614 NCT04986748	NCT05338073 NCT03896958 NCT03358628 NCT05024734

As shown in Table 1, the large diversity of organoid types is matched with a variety of emerging applications in abundant disease contexts (COPD, type 1 diabetes, and psychiatric disorders, among others). Optimistically, the success of these trials in reaching their primary outcomes, could result in organoid technologies becoming a reality in routine clinical care.

### Disease prognosis

The ability of organoids to partly capture a patient phenotype has been naturally explored to yield diagnostic applications. In this way, the linkage of organoid function to a particular disease phenotype may offer possibilities that would otherwise not be possible or unethical, such as the susceptibility to bacterial infection in intestinal organoids, or to respiratory virus infections using lung organoids. Making use of the well-described intestinal organoids, researchers have explored applications in autoimmune disease contexts, such as inflammatory bowel diseases. In an ongoing clinical trial (NCT02874365), researchers plan to morphologically characterize organoids derived from Crohn’s disease and ulcerative colitis patients, as well as healthy controls. By measuring gene expression and protein levels of relevant molecular pathways (Wnt/APC/beta-catenin), genes of tumor initiation (PTEN, BMPR1A, p53 and KRAS), and inflammatory parameters (cytokines and lipid mediators), the group aims to further understand intestinal renewal. However, despite being promising, most of this type of research has been observational rather than diagnostic. Using similar intestinal organoid models, other research groups have ventured into diagnostic applications such as the identification of food allergies and hypersensitivities through insult of intestinal organoids with known food allergens (NCT05056610, NCT05259826), or susceptibility and possible treatments for acute radiation enteritis (NCT05425901).

Novel uses for other tissue-type organoids includes applications as diverse as the study of genetic variants associated with psychiatric disorders using iPSCs differentiated into brain organoids (NCT05480826), renal organoids from iPSCs to understand transcriptional profiles of ciliopathy (NCT04874909) or using endometrial organoids to study the basis for infertility in women with recurrent implantation failure and pregnancy loss (NCT04939064).

In oncology, the use of cancer-derived organoids may be useful to determine the properties of the original tumor. For instance, their performance in specialized assays (such as matrix invasion assays), could teach us something about how invasive this cancer type may be in the patient, and be used as a readout linked to metastasis. Beyond prognosis, oncologists are mostly interested in how patient-derived cancer organoids or tumoroids may respond to a particular treatment such as chemotherapy or radiation. In theory, this information could prove useful in guiding practitioners to develop a particularly relevant therapeutic strategy. This type of interventions that aim to predict treatment outcome and guide therapy will be discussed next.

### Treatment response prediction

As described above, treatment response prediction is a prevalent subset of the use of organoids in personalized medicine. Indeed, there are many examples of how organoids can provide treatment prediction for a variety of cancers such as colorectal (NCT05883683, NCT05832398, NCT05352165, NCT04220242), lung (NCT03979170, NCT04859166, NCT03453307), and pancreatic (NCT03544255, NCT05196334, NCT05927298) cancers. In this section, we highlight some examples of how organoid technology can be leveraged to guide treatment prediction defined as the prediction of treatment response and/or the use of organoids for drug screenings.

For instance, the FORESEE trial (NCT04450706), sponsored by the University of Utah, enrolls patients with metastatic breast cancer to provide personalized genomic and drug sensitivity information to patients. The study utilizes participant’s samples for genome sequencing and organoid-based drug screening. The results are then returned to the clinician which may alter the previously uninformed treatment regimen.

In a different breast cancer trial, the TRIPLEX trial (NCT05404321) sponsored by Centre Francois Baclesse, collects blood and biopsy samples from triple-negative breast cancer patients to develop functional tests of predictive biomarkers for drug responses. In this study, the blood and tumor biopsy samples will be used to generate patient-derived tumor organoids with autologous immune cells, named iPDTO, to evaluate treatment response.^31^ This shows the potential of increasing the complexity of organoid models leading to better modelling of outcome prediction.

The versatility of organoids also allows for the use of treatment prediction in other modalities of cancer therapy, such as radiotherapy (Table 2). For instance, an observational trial sponsored by Maastricht Radiation Oncology, biobanked primary lung cancer organoids and tested the use of hypoxia-activated prodrugs in combination with radiation (NCT04859166). At the same time another observational trial sponsored by Fudan University is validating the use of organoids as a diagnostic tool for chemoradiation sensitivity of rectal cancer (NCT03577808).

**Table 2.** Clinical trials using patient-derived organoids to study chemoradiosensitivity.

Tumor type	Treatment	Study	Ref
Rectal cancer	Radiation	Systemic neoadjuvant and adjuvant control by precision medicine in rectal cancer	NCT04842006
	Chemoradiation	Organoids in predicting chemoradiation sensitivity on rectal cancer	NCT0357780
Liver cancer	Radiation	Stereotactic body radiation therapy for unresectable perihilar cholangiocarcinoma	NCT03307538
Kidney cancer	Proton irradiation	Treatment of newly diagnosed patient’s with wilm’s tumor requiring abdominal radiation delivered with proton beam irradiation	NCT04968990
Brain cancer	Chemoradiation	Patient-derived glioma stem cell organoids	NCT04868396
Esophageal cancer	Chemoradiation	Organoid based response prediction in esophageal cancer	NCT03283527
Lung cancer	Combinatorial regimes	A trial with chemotherapy, immunotherapy, and radiotherapy for patients with newly diagnosed stage IV small cell lung cancer	NCT04951115
	Irradiation	Lung cancer organoids and patient derived tumor xenografts	NCT0509200
	Chemoradiation/hypoxia	Prospective primary human lung cancer organoids to predict treatment response	NCT04859166
Breast cancer	Chemotherapy	A pilot study of a micro-organosphere drug screen platform to lead care in advanced breast cancer	NCT04655573

Aside from cancer, organoids can offer treatment advice for genetic diseases. The multi-center HIT-CF trial (Netherlands Trial Registry, NTR7520) is sponsored by the University Medical Center Utrecht and attempts to assess drug candidates in rectal organoids of cystic fibrosis patients. Cystic fibrosis (CF) is a genetic disease triggered by the loss of function of the *CFTR* gene, resulting in thick secretions in organs such as the lungs. The study aims to gather sufficient data to create an organoid-based test to deliver personalized treatment of CFTR modulators to cystic fibrosis patients.^32^ As the use of organoids for CF treatment prediction is well established, it is possible to create a multi-centered study. However, most organoid studies in other diseases remain localized to each institute. This is partly attributed to the lack of standardization of organoid generation and culture protocols.

### Transplantation or cell-based therapies

Adult-stem cell-derived organoid models rely on fine-tuned tissue culture protocols that enable the ex vivo isolation and expansion of a patient’s stem cells. The individualized nature of patient-derived normal tissue organoids allows their use as autologous sources of transplantation material not limited by histocompatibility, and therefore with less or no fear of rejection. An increasing amount of preclinical evidence is paving the way for clinical trials making use of organoid-technologies for a variety of cell-based therapies. These studies have shown that organoids may mature and integrate in the host tissue when transplanted in animal models.^33^ Clinical applications include the use of intestinal organoids for ulcerative colitis (UMIN000030117, Japanese Registry), encapsulated pancreatic endoderm cells for diabetes patients (NCT03163511), and the use of salivary gland organoids for radiation-induced xerostomia (NCT04593589). The latter trial, from our group (NCT04593589), proposes the prophylactic bio-banking of tissue-dispersed cells derived from otherwise discarded submandibular salivary gland tissue prior to patients with head-and-neck cancer undergoing radiotherapy. After a patient completes their radiotherapy regimen, the salivary gland cells are thawed and cultured into organoids whereafter the patient may undergo autologous salivary gland stem cell-transplantation. This approach may offer a viable alternative for patients that would otherwise face irreparable radiation damage, without hampering tumor control, and has the potential for application in other tissue contexts.

It is noteworthy that clinical trials making use of organoid-technologies do not necessarily transplant organoids into the patient, but may instead be reduced to clumps of cells, or single cells, and have a subpopulation transplanted back into the patient. In this way, organoid cultures enable the expansion of (stem) cell types that would otherwise be impossible to propagate. In oncology, such an approach is being tested using patient-derived tumoroids to screen for tumor-infiltrating lymphocytes and peripheral T cells from the same patient. Tumor-responsive T cells are further selected and monoclonally expanded for the possibility to transfer them back into the patient (NCT03778814). This methodology may translate into improved outcomes for patients with immunocompromised cancer but remains to be proven successful.

Beyond oncology, because organoids maintain the genetic specificity of the source tissue,^21^ the conjunction of organoid technology with gene-editing technologies may provide clinically relevant alternatives for genetic diseases. Although genetic diseases are often caused by multiple gene mutations, early steps have been taken for tackling single-gene hereditary defects, such as cystic fibrosis, an autosomal recessive hereditary disease caused by mutations in the cystic fibrosis transmembrane conductor receptor (CFTR). In 2013, Schwank et al explored this theoretical basis by using CRISPR-Cas9 gene-editing to correct a CFTR mutation in using intestinal organoids derived from patients with cystic fibrosis.^34^ Although gene correction in other tissue types has been explored, and engraftment proven in preclinical animal models (discussed in Ref. ^35^), its use in the clinic remains to be tested.

Genetic engineering to correct defective genes in cells intended for transplantation presents itself with added layers of complexity. First, the genetic templates and the constructs used for correction must be tailored to the individual and their specific allelic presentation. Second, the genetic stability of the modified cells must be warranted, and the risk for unwanted mutations needs to be minimized. Despite promising, these additional steps render genetically engineered cell-therapies labor intensive, costly, and mostly unattainable.

An alternative to bypass genetic engineering is the use of allogeneic cells from matched donors. Recently, researchers in Kobe (Japan) explored the use of allogeneic iPSC-derived retinal organoid sheets in 2 individuals with retinitis pigmentosa (jRCTa050200027,^36^). Retinitis pigmentosa is a hereditary degenerative disease, with over 70 genes reported as causative. Individuals with retinitis pigmentosa suffer from a progressive degeneration of photoreceptors, resulting in an increasing loss of vision. The single-arm, uncontrolled, open clinical study, resulted in stable engraftment at 2 years follow-up, with increased retinal thickness at the transplant site and less progressive changes in visual function than those of the untreated eye during the follow-up. Importantly, no serious adverse events were reported in either subject. Despite the obvious limitations in patient number (2) and the lack of study blinding, this pilot study provides initial evidence for the use of transplantable iPSC-derived organoids as a potential therapeutic approach. Another example yet to reach the clinical trial stage is the use of inner ear organoids to restore auditory loss,^37,38^ with encouraging results of their transplantation in a rodent model, but no evidence in humans. Additional organoid types derived from iPSCs have been described but appear further from reaching the clinical trial stage.

## Limitations and future perspectives

While organoid technology holds promise to deliver personalized medicine, there remain a few challenges to be addressed. First, a highly specialized protocol with a complex culture medium for each type of organoids is currently needed to establish organoids. This provides an entry barrier for many laboratories to establish their own organoid cultures. Meanwhile, different laboratories working with the same types of organoids, often use different protocols, extracellular matrices, and culture media to establish their organoids. The lack of organoid model standardization makes it difficult for conclusions to be reproduced, and to effectively deliver personalized medicine in the clinic. Therefore, future efforts need to be focused on developing standardized protocols for establishing tissue type-specific organoids.

Secondly, the tissue used to establish organoids is very important in the development of organoids. In gastrointestinal cancer, it is not uncommon to face microbial contaminations while establishing organoids,^39^ or for other cancer type organoid cultures to be overgrown by normal tissue organoids.^40^ Additionally, for the establishment of normal organoids, the location of tissue sampling is highly critical. In salivary gland organoids, the site from which the original biopsy is derived may largely influence the number of stem/progenitor cells.^41^ In the future, large amounts of data still need to be gathered to be able to devise the ideal tissue collection protocols optimized for each organoid category.

Thirdly, as discussed above, organoids can only derive cell types based on the differentiation capacity of the stem cells present in the tissue of origin. In turn, this means that organoids do not contain supporting systems such as vasculature and immune components, thus reducing their predictive value. Therefore, recent developments of more complex culture systems such as the spatially controlled bio printing of assembloids,^42^ and organ- on-chip models, can represent higher fidelity models if or when needed. While assembloids are complex organoids containing cell types derived from multiple lineages or by fusion of different organoids, organ-on-chip models utilize microfluidic culture devices to replicate microphysiological systems such as dynamic flow.^43^ In this way, the integration of new technologies may further improve the predictive power of existing models and fuel potential novel applications.

## Data Availability

No new data were generated or analyzed in support of this research.

## References

[1] Goetz LH , Schork NJ. Personalized medicine: motivation, challenges, and progress. Fertil Steril. 2018;109(6):952-963. 10.1016/j.fertnstert.2018.05.006 29935653 PMC6366451

[2] Sato T , Vries RG, Snippert HJ, et al. Single Lgr5 stem cells build crypt-villus structures in vitro without a mesenchymal niche. Nature. 2009;459(7244):262-265. 10.1038/nature07935 19329995

[3] Sachs N , de Ligt J, Kopper O, et al. A living biobank of breast cancer organoids captures disease heterogeneity. Cell. 2018;172(1-2):373-386.e10. 10.1016/j.cell.2017.11.010 29224780

[4] Ouchi R , Togo S, Kimura M, et al. Modeling steatohepatitis in humans with pluripotent stem cell-derived organoids. Cell Metab. 2019;30(2):374-384.e6. 10.1016/j.cmet.2019.05.007 31155493 PMC6687537

[5] Gómez-Mariano G , Matamala N, Martínez S, et al. Liver organoids reproduce alpha-1 antitrypsin deficiency-related liver disease. Hepatol Int. 2020;14(1):127-137. 10.1007/s12072-019-10007-y 31832977 PMC6994530

[6] Pringle S , et al. Salivary gland stem cells age prematurely in primary Sjögren’s syndrome. Arthritis Rheumatol. 2019;71(1):133-142. 29984480 10.1002/art.40659 PMC6607019

[7] Montenegro-Miranda PS , van der Meer JHM, Jones C, et al. A novel organoid model of damage and repair identifies HNF4α as a critical regulator of intestinal epithelial regeneration. Cell Mol Gastroenterol Hepatol. 2020;10(2):209-223. 10.1016/j.jcmgh.2020.02.007 32145468 PMC7301200

[8] Birey F , Andersen J, Makinson CD, et al. Assembly of functionally integrated human forebrain spheroids. Nature 2017;545(7652):54-59. 10.1038/nature22330 28445465 PMC5805137

[9] Workman MJ , Mahe MM, Trisno S, et al. Engineered human pluripotent-stem-cell-derived intestinal tissues with a functional enteric nervous system. Nat Med. 2017;23(1):49-59. 10.1038/nm.4233 27869805 PMC5562951

[10] Soto-Gamez A , van Es M, Hageman E, et al. Mesenchymal stem cell-derived HGF attenuates radiation-induced senescence in salivary glands via compensatory proliferation. Radiother Oncol. 2024;190:109984. 10.1016/j.radonc.2023.109984 37926332

[11] Khedoe P , et al. Cigarette smoke restricts the ability of mesenchymal cells to support lung epithelial organoid formation. Front Cell Dev Biol. 2023;11:1165581. 37795260 10.3389/fcell.2023.1165581 PMC10546195

[12] Wieser RJ , Oesch F. Contact inhibition of growth of human diploid fibroblasts by immobilized plasma membrane glycoproteins. J Cell Biol. 1986;103(2):361-367. 10.1083/jcb.103.2.361 3733871 PMC2113841

[13] Horton C , Liu Y, Yu C, Xie Q, Wang ZA. Luminal-contact-inhibition of epithelial basal stem cell multipotency in prostate organogenesis and homeostasis. Biol Open 2019;8(10):bio045724. 10.1242/bio.045724 31540905 PMC6826291

[14] Heo JH , Kang D, Seo SJ, Jin Y. Engineering the extracellular matrix for organoid culture. Int J Stem Cells 2022;15(1):60-69. 10.15283/ijsc21190 35220292 PMC8889330

[15] Co JY , Margalef-Català M, Monack DM, Amieva MR. Controlling the polarity of human gastrointestinal organoids to investigate epithelial biology and infectious diseases. Nat Protoc. 2021;16(11):5171-5192. 10.1038/s41596-021-00607-0 34663962 PMC8841224

[16] Kleinman HK , McGarvey ML, Hassell JR, et al. Basement membrane complexes with biological activity. Biochemistry 1986;25(2):312-318. 10.1021/bi00350a005 2937447

[17] Yui S , Azzolin L, Maimets M, et al. YAP/TAZ-Dependent reprogramming of colonic epithelium links ECM remodeling to tissue regeneration. Cell Stem Cell 2018;22(1):35-49.e7. 10.1016/j.stem.2017.11.001 29249464 PMC5766831

[18] Kozlowski MT , Crook CJ, Ku HT. Towards organoid culture without Matrigel. Commun Biol. 2021;4(1):1387. 10.1038/s42003-021-02910-8 34893703 PMC8664924

[19] Broguiere N , Isenmann L, Hirt C, et al. Growth of epithelial organoids in a defined hydrogel. Adv Mater. 2018;30(43):e1801621. 10.1002/adma.201801621 30203567

[20] Schaafsma P , Kracht L, Baanstra M, Jellema-de Bruin AL, Coppes RP. Role of immediate early genes in the development of salivary gland organoids in polyisocyanopeptide hydrogels. Front Mol Biosci. 2023;10:1100541. 10.3389/fmolb.2023.1100541 36818041 PMC9932530

[21] Bergenheim F , Fregni G, Buchanan CF, et al. A fully defined 3D matrix for ex vivo expansion of human colonic organoids from biopsy tissue. Biomaterials 2020;262:120248. 10.1016/j.biomaterials.2020.120248 32891909

[22] Hernandez-Gordillo V , Kassis T, Lampejo A, et al. Fully synthetic matrices for in vitro culture of primary human intestinal enteroids and endometrial organoids. Biomaterials 2020;254:120125. 10.1016/j.biomaterials.2020.120125 32502894 PMC8005336

[23] Takahashi K , Tanabe K, Ohnuki M, et al. Induction of pluripotent stem cells from adult human fibroblasts by defined factors. Cell 2007;131(5):861-872. 10.1016/j.cell.2007.11.019 18035408

[24] Kim J , Koo BK, Knoblich JA. Human organoids: model systems for human biology and medicine. Nat Rev Mol Cell Biol. 2020;21(10):571-584. 10.1038/s41580-020-0259-3 32636524 PMC7339799

[25] Xu H , Jiao D, Liu A, Wu K. Tumor organoids: applications in cancer modeling and potentials in precision medicine. J Hematol Oncol. 2022;15(1):58. 10.1186/s13045-022-01278-4 35551634 PMC9103066

[26] Zhou Z , Cong L, Cong X. Patient-derived organoids in precision medicine: drug screening, organoid-on-a-chip and living organoid biobank. Front Oncol. 2021;11:762184. 10.3389/fonc.2021.762184 35036354 PMC8755639

[27] Wang Y , Li Y, Sheng Z, et al. Advances of patient-derived organoids in personalized radiotherapy. Front Oncol. 2022;12:888416. 10.3389/fonc.2022.888416 35574360 PMC9102799

[28] Gao D , Vela I, Sboner A, et al. Organoid cultures derived from patients with advanced prostate cancer. Cell 2014;159(1):176-187. 10.1016/j.cell.2014.08.016 25201530 PMC4237931

[29] van de Wetering M , Francies HE, Francis JM, et al. Prospective derivation of a living organoid biobank of colorectal cancer patients. Cell 2015;161(4):933-945. 10.1016/j.cell.2015.03.053 25957691 PMC6428276

[30] Xie X , Li X, Song W. Tumor organoid biobank-new platform for medical research. Sci Rep. 2023;13(1):1819. 10.1038/s41598-023-29065-2 36725963 PMC9892604

[31] Divoux J , Florent R, Jacobs M, et al. The TRIPLEX study: use of patient-derived tumor organoids as an innovative tool for precision medicine in triple-negative breast cancer. BMC Cancer 2023;23(1):883. 10.1186/s12885-023-11362-8 37726786 PMC10508010

[32] van Mourik P , Michel S, Vonk AM, et al. Rationale and design of the HIT-CF organoid study: stratifying cystic fibrosis patients based on intestinal organoid response to different CFTR-modulators. Transl Med Commun. 2020;5(1):9.

[33] Pringle S , Maimets M, van der Zwaag M, et al. Human salivary gland stem cells functionally restore radiation damaged salivary glands. Stem Cells. 2016;34(3):640-652. 10.1002/stem.2278 26887347

[34] Schwank G , Koo B-K, Sasselli V, et al. Functional repair of CFTR by CRISPR/Cas9 in intestinal stem cell organoids of cystic fibrosis patients. Cell Stem Cell 2013;13(6):653-658. 10.1016/j.stem.2013.11.002 24315439

[35] Huang Y , Huang Z, Tang Z, et al. Research progress, challenges, and breakthroughs of organoids as disease models. Front Cell Dev Biol. 2021;9:740574. 10.3389/fcell.2021.740574 34869324 PMC8635113

[36] Hirami Y , Mandai M, Sugita S, et al. Safety and stable survival of stem-cell-derived retinal organoid for 2 years in patients with retinitis pigmentosa. Cell Stem Cell 2023;30(12):1585-1596.e6. 10.1016/j.stem.2023.11.004 38065067

[37] Chen W , Jongkamonwiwat N, Abbas L, et al. Restoration of auditory evoked responses by human ES-cell-derived otic progenitors. Nature 2012;490(7419):278-282. 10.1038/nature11415 22972191 PMC3480718

[38] Koehler KR , Nie J, Longworth-Mills E, et al. Generation of inner ear organoids containing functional hair cells from human pluripotent stem cells. Nat Biotechnol. 2017;35(6):583-589. 10.1038/nbt.3840 28459451 PMC5462862

[39] Nam SY , Lee SJ, Lim HJ, Park JY, Jeon SW. Clinical risk factors and pattern of initial fungal contamination in endoscopic biopsy-derived gastrointestinal cancer organoid culture. Korean J Intern Med. 2021;36(4):878-887. 10.3904/kjim.2020.474 33826841 PMC8273816

[40] Dijkstra KK , Monkhorst K, Schipper LJ, et al. Challenges in Establishing Pure Lung Cancer Organoids Limit Their Utility for Personalized Medicine. Cell Rep. 2020;31(5):107588. 10.1016/j.celrep.2020.107588 32375033

[41] van Luijk P , et al. Sparing the region of the salivary gland containing stem cells preserves saliva production after radiotherapy for head and neck cancer. Sci Transl Med. 2015;7(305):305ra-30147. 10.1126/scitranslmed.aac4441 PMC4964284 26378247

[42] Roth JG , Brunel LG, Huang MS, et al. Spatially controlled construction of assembloids using bioprinting. Nat Commun. 2023;14(1):4346. 10.1038/s41467-023-40006-5 37468483 PMC10356773

[43] Ingber DE. Human organs-on-chips for disease modelling, drug development and personalized medicine. Nat Rev Genet. 2022; 23(8):467-491. 10.1038/s41576-022-00466-9 35338360 PMC8951665

